# Warts

**Published:** 1872-07

**Authors:** 


### WARTS

Are a species of "corn," growing from the surface of the skin, from superficial roots, of very little vitality. They may be removed with the knife, but not by mere shaving them off, like a common weed; they must be cut up by the roots. Numerous cases are given in medical works, where cutting has been followed by bleedings which could not be stopped until large quantities of blood flowed, almost endangering life. If the very topmost part is sliced off, and the wart has rubbed into it a soft stick dipped in aquafortis, repeated daily for a week or more, if necessary, the wart will disappear.

Lunar caustic sometimes accomplishes the same object.

A milder method is, after shaving off the top, paint them twice a day with a camel's-hair pencil dipped in common coal-oil kerosene. They sometimes disappear after a week or two's application, leaving no mark or scar whatever. The cure will be expedited by saturating a bit of cotton or lint, or soft rag, a little larger than the wart; lay it on, and a piece of oiled silk over that, around and larger than the cotton, and bind it on so as to keep it in its place. Do this night and morning. The oil either absorbs the wart, or, more likely, destroys the vitality of the roots, and the wart dies, as a plant would when its roots are destroyed.

Creosote applied to a wart, then covered with sticking-plaster, repeated every third day for two or three weeks, has removed the most obstinate warts. Warts have been removed permanently and without much pain, as they have but little sensibility, by tying a silk thread or horse hair around the neck of the protuberance, as some of them grow up in a mushroom shape.

Sometimes warts disappear without anything being done for them. Hence, anything done for a wart, the last thing done, although nature was about removing it, has the credit of the cure. Thus many things are said to be efficacious which really have no curative effect whatever.

Warts appearing in more private parts of the body, are sometimes cured by applying a powder made of savin leaves and verdigris.

Pass a needle through a wart, apply a flame to one end of the needle, until the wart begins to fry; it will disappear, never to return. But the better plan, as above stated, is to dip the end of a knitting-needle in aquafortis, and rub it on the head of a wart; rub it in well, or use coal oil. But in using nitric acid, or nitrate of silver, avoid letting it touch the skin around the wart.

